# Impact of COVID-19 on serum melatonin levels and sleep parameters in children

**DOI:** 10.3906/sag-2012-361

**Published:** 2021-08-30

**Authors:** Özlem YAYICI KÖKEN, Pembe GÜLTUTAN, Merve Sibel GÜNGÖREN, Gülsüm İclal BAYHAN, Deniz YILMAZ, Esra GÜRKAŞ, Hamit ÖZYÜREK, Ayşegül Neşe ÇITAK KURT

**Affiliations:** 1 Department of Pediatric Neurology, Children’s Hospital, Ankara City Hospital, Ankara Turkey; 2 Düzen Laboratories Group, Ankara Turkey; 3 Department of Pediatric Infectious Disease, Children’s Hospital, Ankara City Hospital, Ankara Turkey; 4 Department of Pediatric Neurology, Yıldırım Beyazıt University, Faculty of Medicine, Ankara Turkey

**Keywords:** Melatonin sleep, pediatric, COVID-19, children

## Abstract

**Background/aim:**

This study aimed to analyze the serum melatonin levels and changes in sleep patterns in pediatric patients with coronavirus disease 2019 (COVID-19).

**Materials and methods:**

This study was designed as a descriptive, cross-sectional study. Serum melatonin levels and sleep parameters of children with the diagnosis of COVID-19 who had mild and moderate disease (i.e., COVID-19 group) were compared with those of children admitted with non-COVID-19 nonspecific upper respiratory tract infection (i.e., control group). The sleep disturbance scale for children (SDSC) questionnaire was applied to the participants› primary caregivers to analyze their sleep patterns at present and six months before symptom onset and to investigate the impact of COVID-19 on sleep patterns.

**Results:**

The entire study cohort consisted of 106 patients. The COVID-19 group included 80 patients, while the control group consisted of 26 patients. The mean serum melatonin levels were 136.72 pg/mL and 172.63 pg/mL in the COVID-19 and control groups, respectively (p = 0.16). There was no significant difference between the groups in terms of 6 subcategories of the SDSC questionnaire regarding the present time and 6 months before symptom onset. The total SDSC scores were also similar in two different evaluation time points described above (p = 0.99).

**Conclusions:**

We conclude that COVID-19 did not impact the sleep parameters of children. Serum melatonin levels of all patients were higher than the reference range; however, they were higher in the non-COVID-19 patient group than the COVID-19 group. Since serum melatonin levels were higher than the reference values in children with COVID-19, and this disease is significantly less morbid in children, melatonin may have protective effects against COVID-19.

## 1. Introduction

Melatonin is a hormone secreted by the pineal gland, and it is the primary regulator of sleep^1^. Although it is known that melatonin has antiinflammatory, antioxidant, and immunomodulatory functions during infection with bacteria and viruses, its specific role in the novel coronavirus disease 2019 (COVID-19) has not been widely investigated [1]. To date, data obtained regarding COVID-19 infection showed significantly lower morbidity and mortality rates in the young patient population and children [1–3]. This finding implies that patient age may have a critical role in this disease’s pathogenesis. Since the serum concentration of melatonin is inversely correlated with age, it would not be unreasonable to investigate the potential role of melatonin in the pathogenesis and progress of COVID-19 [1–2,4]. 

Photosensitive retinal-containing receptors mediate the release of melatonin from the pineal gland [4].Thus, its secretion is inhibited during daylight. It is known that COVID-19 is a zoonotic disease, and bats are the natural carriers of coronavirus [4]. Bats are nocturnal hunters; they avoid daylight and hide in dark caves during the daytime. As a result, they continuously have high levels of melatonin compared to the human. Coronavirus infection is not fatal in bats with minimal or no symptoms. Despite these facts, the alleviating role of melatonin hormone or relatively high serum melatonin concentrations in the young patient population and children with COVID-19 has not been investigated.

This study aimed to analyze the protective role of melatonin in children with COVID-19 infection. Also, we aimed to investigate these children’s sleep patterns to interrogate the relationships between melatonin levels and sleep patterns and the impact of COVID-19 on the sleep patterns of these patients.

## 2. Materials and methods

**Figure 1:**
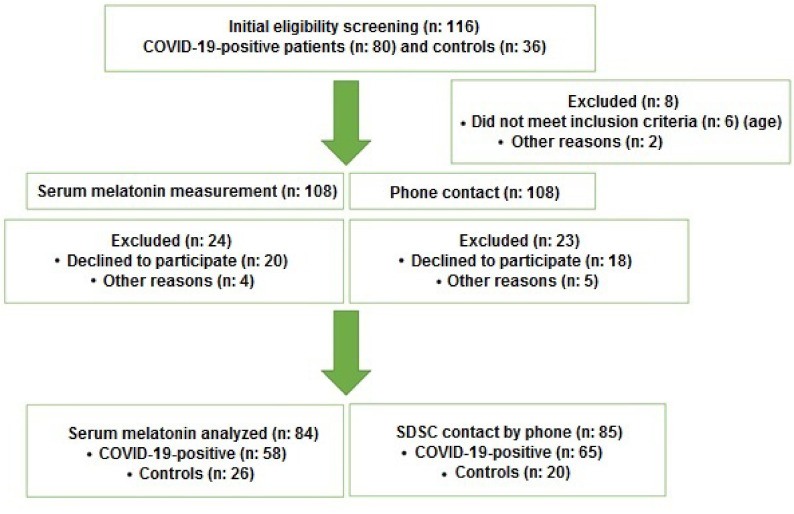
Flowchart of the study.

This study was approved by the Ethical Review Committee of Ankara City Hospital (06.06.2020/E1-20-564). Pediatric patients admitted to Ankara City Hospital, Pediatric Health Center after confirmation of COVID-19 infection between June 2020 and August 2020 by at least two positive PCR test results, were included in this prospective study. Children hospitalized at the same center with the clinical suspicion of COVID-19 but had at least two negative PCR test results constituted the control group. These control group patients presented with high fever and other nonspecific upper respiratory tract infection symptoms without an apparent history of contact with known COVID-19 cases. The age range of the target population was 6–16 years since the sleep disturbance scale for children (SDSC) questionnaire assesses children and adolescents in this age group [5,6]. Patients with a history of previous COVID-19 infection, history of melatonin treatment in the previous 4 weeks, sleep apnea syndrome, chronic neuromuscular disease, and those whose legal guardians did not give consent were excluded. The study participant flowchart is shown in Figure 1. The COVID-19-positive patients were classified according to disease severity based on clinical and laboratory data [7]. Patients with no clinical symptoms or findings and normal chest imaging results were classified as asymptomatic, while those who had a high fever (i.e., a temperature higher than 37.8 °C), myalgia, throat pain, coughing, or nasal discharge with normal findings of lung auscultation were classified as a mild disease. On the other hand, patients who had a high fever, coughing, or wheezing accompanied by pneumonia or those who had COVID-19-associated changes in their computerized chest tomography without clinical findings were classified as a moderate disease. Patients who had dyspnea and low oxygen saturation (i.e., < 92%) were classified as severe, and those who had rapidly progressing acute respiratory distress syndrome or respiratory failure with multiple organ dysfunction were classified as critical. 

Waldhauser et al. analyzed the alterations in serum melatonin levels with growing and aging [2]. The reported values in this study were used as references of serum melatonin values. These authors reported that the mean serum melatonin levels were 160 pg/mL for the 6–7 age group, 110 pg/mL for the 7–12 age group, 85 pg/mL for the 12–15 age group, and 55 pg/mL for patients older than 15. 

### 2.1. Sample collection

Serum samples were collected from all patients on the day of admission and within three days of their first symptoms for analyzing the melatonin levels. They were collected between 06:00 and 07:00 am in the dim light of less than 30 lux (i.e., standard hospital lighting) from the patients after overnight fasting. Postural conditions and environmental lighting were the same for all patients during venipuncture. First, 2 mL of blood was drawn from the antecubital vein and subsequently centrifuged. The serum was kept at –20 °C. All study participants went to bed between 21:30 and 22:30 and woke up between 07:00 and 08:00 am. Serum melatonin levels were analyzed using the ELISA method (Human Melatonin ELISA kit, Catalogue Number: E1013Hu, Bioassay Technology Laboratory, Shanghai, China). 

### 2.2. Sleep disturbance scale for children

The SDSC questionnaire was applied to the primary caregivers by the same researcher (P.G.) on the phone. The primary caregiver was requested first to answer the questions considering the last week, which covered disease onset (i.e., present time) and second by considering the six months before infection (i.e., 6 months before symptom onset). The primary reason for the evaluation of sleep at two different time frames was analyzing the impact of COVID-19 infection on the patients’ sleep characteristics and comparison of the patients and controls in this regard. This questionnaire containing 27 Likert-type questions was designed to evaluate sleep disturbances in children and adolescents and was validated for the Turkish language [5,6]. It evaluates subsets of sleep disturbances, namely, disorders of initiating and maintaining sleep, sleep breathing disorders, disorders of arousal, sleep-wake transition disorders, disorders of excessive somnolence, and sleep hyperhidrosis) in children and adolescents. The cut-off point is 39 on this scale; points higher than this are associated with sleep disturbances [5]. A reliability test has been performed in order to test the data set obtained from the questionnaire forms. Alpha coefficient (Cronbach’s alpha) has been used for reliability analysis. Sleep disorders in children have been measured using a questionnaire consisting of 24 questions and the reliability of the sleep disorder scale was found to be 0.840 for the last 6 months and 0.825 for the last week. 

### 2.3. Statistical analysis

Data analyses were performed using IBM SPSS Statistics v. 23.0 (Armonk, NY, USA). Whether the distribution of continuous variables was normal or not was determined by the Kolmogorov–Smirnov test. The Levene test was used for the evaluation of the homogeneity of the variances. Unless specified otherwise, continuous variables were presented as means± standard deviations (SD) for normally distributed data, and medians and ranges (i.e., minimum and maximum values) for data with skewed distribution. Categorical variables were given as the numbers (n) or percentages (%). The student’s t test was used to compare continuous normally distributed variables between two independent groups, while the Mann–Whitney U test was implemented for comparing the nonnormally distributed data. Categorical variables were compared using the Pearson chi-square test or Fisher exact test. Correlations between variables were evaluated using the Pearson or Spearman correlation analysis. A p value of less than 0.05 was considered significant.

## 3. Results

**Figure 2:**
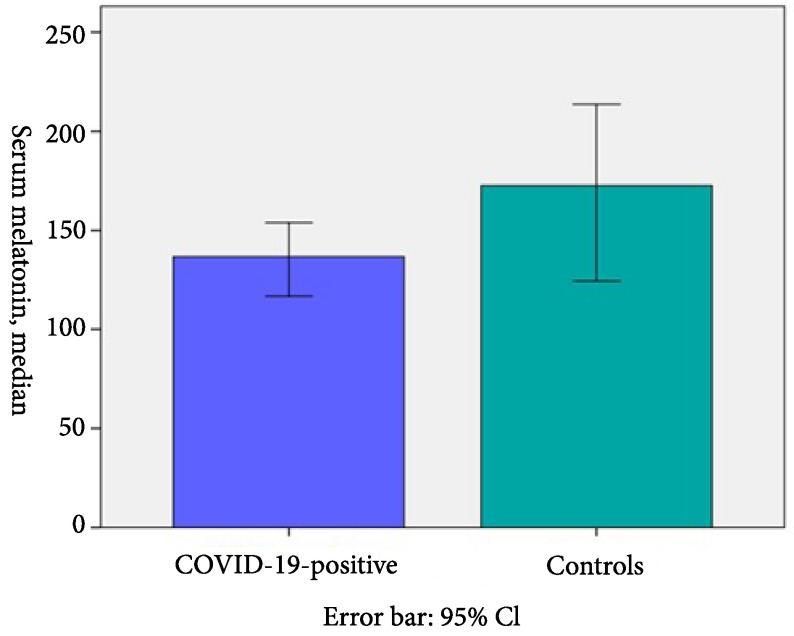
Serum melatonin levels based on COVID-19 positive and control group.

The target population of this study included 118 patients. Five patients who refused to give blood samples, 5 patients whose primary caregivers could not provide the authors with reliable data, and 2 patients with inconclusive laboratory data were excluded. Thus, the entire study cohort consisted of 116 patients. Among these patients, 58 were COVID-19 patients, while 26 patients were assigned to the control group for measuring serum melatonin levels, and 65 were COVID-19 patients, while 20 patients were assigned to the control group for applying SDSC (Figure 1). All COVID-19 patients had either mild (85%) or moderate disease (15%) based on the classification. Demographic data of the patients and serum melatonin levels are shown in Table 1. Comparative analysis revealed that serum melatonin levels were relatively lower in the COVID-19 patient group than the control group. However, the difference was not statistically significant (Table 1, Figure 2).

**Table 1:** Age, sex, and serum melatonin levels of the participants.

		COVID-19-positive (n: 58)	COVID-19-negative (n: 26)	P-value
Sex (n,%)	Male	34 (58.6%)	20 (76.9%)	0.11
	Female	24 (41.4%)	6 (23.1%)	
Age (mo, mean ± SD)		156.42 ± 42.33	155.12 ± 38.30	0.13
Serum melatonin (pg/mL, median, min-max)		136.72 (48.09–1217.13)	172.63 (59.16–1171.09)	0.16

The differences in serum melatonin levels were evaluated by grouping the participants based on their age and corresponding reference levels, as shown in Table 2. There were 5 patients in the 6–7-year-old age group. However, since there were no control group patients in this age group, they were not included in Table 2. The serum melatonin levels were higher than the age-referenced values in both patient groups of our study (Table 3) [2]. Comparison of the serum melatonin levels based on patient age elucidated that the COVID-19 patients had lower serum melatonin levels than the patients in the control group in all age subgroups. Although serum melatonin levels of our COVID-19 patients were lower than control group patients in all age groups, a statistically significant difference was detected only in the 7–11 age group (p = 0.03). All study participants, except for the COVID-19 patients in the 7–12 age group, had significantly higher serum melatonin levels than the reference values (Table 3). 

**Table 2:** Comparison of the serum melatonin levels based on age.

Age groups	Serum melatonin levels (n: 84) (median, min-max)		P-value
	COVID-19-positive(n: 58)	COVID-19-negative(n: 26)	
7–12 years (n: 30)	119.33 (65.30–370.31)	182.68 (85.14–1171.09)	0.03
12–15 years (n: 25)	116.86 (69.64–849.81)	137.39 (59.16–213.75)	0.99
15 years or older (n: 24)	139.21 (48.09–1217.13)	172.63 (133.41–227.83)	0.79

**Table 3:** Comparison of melatonin levels between the COVID-positive patients and the control group based on age (age-based reference values have been added from reference 2).

Age	Reference values(pg/mL)	Serum melatonin levels (n: 84) (median, min-max)		P-value1,2*
		COVID-19-positive(n: 58)	COVID-19-negative(n: 26)	
7–12 years(n: 30)	110	(n: 14) 119.33(65.30–370.31)	(n: 16) 182.68(85.14–1171.09)	0.0210.182
12–15 years(n: 25)	85	(n: 19) 116.86(69.64–849.81)	(n: 6) 137.39(59.16–213.75)	0.0210.052
15 years or older (n: 24)	55	(n: 20) 139.21(48.09–1217.13)	(n: 4) 172.63(133.41–227.83)	0.00210.0082

*P value1 indicates to comparison of reference value and non-COVID-19 control group, P value2 indicates comparison of COVID-19 positive group and reference values.

Comparison of sleep parameters regarding 6 months before symptom onset and present time between COVID-19 patients and control group patients are displayed in the Appendix. There was no significant difference regarding the six subsets of sleep disturbances evaluated by SDSC performed based on 6 months before symptom onset and current data. The changes in the SDSC subset scores and total scores are displayed in Table 4. 

**Table 4:** Summary of the temporal changes in the SDSC subcategories of COVID-19 positive group.

COVID-19 positive		6 months prior to onset	Onset of infection	Change
Total score	r	–0.031	–0.013	–0.088
	P	0.834	0.927	0.550
Initiating and maintaining sleep	r	–0.038	–0.045	–0.050
	P	0.793	0.759	0.732
Sleep breathing disorders	r	0.203	0.063	–0.072
	P	0.162	0.666	0.623
Disorders of arousal	r	0.038	0.037	–0.082
	P	0.795	0.800	0.577
Sleep-wake transition disorders	r	0.086	0.152	0.204
	P	0.556	0.297	0.159
Excessive somnolence	r	–0.047	–0.065	–0.145
	P	0.746	0.658	0.319
Sleep hyperhidrosis	r	–0.017	–0.064	–0.115
	P	0.908	0.663	0.432

The comparison of both total SDSC scores and SDSC subcategory scores of two time points (i.e., 6 months before symptom onset vs. present time) revealed no statistically significant difference between the two groups (p = 0.99). A review of the SDSC scores of the COVID-19 patients showed that 4 (6.2%) patients had scores higher than 39 (i.e., the cut-off value) in the ‘present time’ evaluation, while 5 (7.7%) patients had scores higher than 39 in the evaluation regarding 6 months before symptom onset. One particular control group patient had SDSC scores higher than 39 in both evaluations regarding the present time and six months before symptom onset.

## 4. Discussion

To the best of our knowledge, this study was the first to investigate the serum melatonin levels and sleep patterns in children with COVID-19. The role of melatonin in COVID-19 is still unclear, as data concerning this disease’s pathophysiology is still accumulating [8]. In the adult and elderly population, melatonin is thought to have beneficial effects providing better clinical outcomes for COVID-19 patients admitted to critical care units [1]. Melatonin exerts these positive effects by reducing vessel permeability, decreasing anxiety and sedation need, and improving sleep quality. Therefore, some researchers advocated its use as adjuvant therapy. In our study, serum melatonin levels were lower in children and adolescents with mild or moderate COVID-19 than the control group patients with nonspecific upper respiratory tract infections. However, the difference was not statistically significant. Also, serum melatonin levels of the children and adolescents in both COVID-19 and control groups were significantly higher than the reference values adjusted for age [2]. This finding leads to the hypothesis that serum melatonin levels increase during the acute phase of all viral infections, including the new type of coronavirus infection. The common denominator of studies investigating the relationship of melatonin with viral, bacterial, and parasitic infections is the expectation of using melatonin to treat these infections [9–11] However, none of these studies were conducted with pediatric patients.

Serum melatonin concentrations start to decline with age after the neonatal period [2]. Contrary to this information and the reference values obtained from healthy children, average melatonin levels of 12–15 years old children were higher than that of patients who were 15 years old and older [2]. Although conflicting data exist, a manuscript published in 1992 reports a minor increase in melatonin levels during the puberty [12]. We compared the melatonin levels between COVID-19 positive patients and COVID-19 negative patients with an infection and did not observe serum melatonin levels showing a positive correlation with age in either group, contrary to data from healthy children. This observation leads to the question of whether there is an age-related difference between healthy and infected children concerning serum melatonin levels however, there is no study investigating this question in the literature. We were not able to test this hypothesis since healthy controls were not included in our study. Crowley et al. have investigated the relationship between light exposure and melatonin secretion from the pineal gland and have compared pre-mid and late pubertal melatonin levels. The authors stated that pre- to mid-pubertal group showed significantly greater melatonin suppression on light exposure but no significant differences were seen between pre-, mid, and late pubertal groups in morning melatonin suppression [13]. This study shows that phases of puberty affect melatonin secretion patterns. This points out to the notion that melatonin secretion as a pro-inflammatory or antiinflammatory response to infection could be different from healthy children, similar to the different levels of suppression caused by light exposure in different stages of puberty.

On the other hand, it is known that COVID-19 usually has a mild course in pediatric patients [14–15]. As such, approximately 90% of pediatric patients who do not have comorbidities usually exhibit mild or moderate symptoms with relatively lower morbidity rates. Detection of high serum melatonin levels in pediatric COVID-19 patients has strengthened the theory that melatonin may have protective effects against the progression of COVID-19. Since there were no severe COVID-19 cases in our study, serum melatonin levels could not be compared between mild or moderate cases and severe cases. 

Lifestyle changes due to the COVID-19 pandemic significantly increased the risk of sleep disorders both in patients and healthy population [16–17]. Some studies investigated the sleep disorders in patients who stayed in self-isolation or who were hospitalized for long periods or those who had a history of long-term admission to the intensive care unit. In one of these studies, the frequency of insomnia was 17.4% in Italian patients quarantined for 14 days [16]. Another study from Taiwan investigated sleep disorders in social media users and reported that the rate of sleep disturbances was 55.8% in this patient population [17]. Also, a French study presented complicated sleep disturbance cases in the young adult population during the pandemic period [18].

It is widely accepted that melatonin prevents sleep deprivation, reduces delirium risk, and shortens the length of stay in patients staying at intensive care units [19]. Our literature did not reveal any studies investigating the impact of COVID-19 on sleep parameters in the pediatric patient population. One of our aims was to investigate the changes in children’s sleep patterns with COVID-19 and compare these changes with the changes detected in a group of children who had nonspecific upper respiratory tract infections by questioning the primary caregivers of the participants. The two groups showed similar results in both evaluations regarding the present time and 6 months before symptom onset. Similarly, no difference was found by comparing the SDSC scores concerning present time and six months before symptom onset. These results demonstrated that COVID-19 did not cause any significant changes in the sleep patterns of children. 

It was reported that melatonin decreased sleep onset latency and prolonged total sleep time [20]. Although the serum melatonin levels of all study participants were higher than the reference values in our study, no difference was detected in sleep parameters related to initiating and maintaining sleep. We aimed to take serum samples under similar temporal and environmental conditions considering that a difference of even 1 h can result in a significant difference in melatonin levels. It is also known that changes in sleep timing or exposure to light in the evening can result in different melatonin onset times, leading to different melatonin levels in the following morning [21]. Therefore, although we aimed to obtain standardized conditions, the wide range observed in the serum melatonin levels might have been caused by the inevitable differences in the conditions. 

Our study has some limitations which need to be considered while evaluating its findings. First, the SDSC questionnaires were performed by phone. Although the optimal method of performing a questionnaire is in-person interviews, we had to conduct them on the phone due to contact isolation. Second, the primary caregiver was expected to give sleep-related information regarding the present time and 6 months before symptom onset. The reliability of the latter can be considered doubtful. Third, since there were no severe COVID-19 cases in our cohort, we could not compare the serum melatonin levels of mild or moderate COVID-19 cases with those of severe cases. This limitation is significant because melatonin is used for adjuvant treatment of some patients with severe COVID-19 who are followed in intensive care units [1].

Our study showed that COVID-19 did not cause any changes in sleep parameters of children. Although we detected that serum melatonin levels of all patients participating in our study were higher than the age-adjusted reference values, they were higher in the non-COVID-19 patient group than the patients diagnosed with COVID-19. This finding does not support our hypothesis; however, serum melatonin levels higher than the reference range in pediatric patients with COVID-19 can be considered as a sign of melatonin’s protective effect in these patients. Nevertheless, our results need to be confirmed by further prospective studies conducted with more extensive patient series. 

## Informed consent

This study was approved by the Ethical Review Committee of Ankara City Hospital (06.06.2020/E1-20-564). 

Written informed consent was obtained from all patients’ parents or legal guardians of the children included.
